# Cathelicidin LL37 Promotes Osteogenic Differentiation *in vitro* and Bone Regeneration *in vivo*

**DOI:** 10.3389/fbioe.2021.638494

**Published:** 2021-05-03

**Authors:** Lunhao Li, Yiyu Peng, Qingyue Yuan, Jing Sun, Ai Zhuang, Xiaoping Bi

**Affiliations:** ^1^Department of Ophthalmology, Shanghai Ninth People’s Hospital, Shanghai Jiao Tong University School of Medicine, Shanghai, China; ^2^Shanghai Key Laboratory of Orbital Diseases and Ocular Oncology, Shanghai, China

**Keywords:** peptide cathelicidin LL37, bone regeneration, osteogenic differentiation, human adipose tissue-derived mesenchyme stem cells, rat calvaria bone defect model

## Abstract

Different types of biomaterials have been used to repair the defect of bony orbit. However, exposure and infections are still critical risks in clinical application. Biomaterials with characteristics of osteogenesis and antibiosis are needed for bone regeneration. In this study, we aimed to characterize the antimicrobial effects of cathelicidin-LL37 and to assess any impacts on osteogenic activity. Furthermore, we attempted to demonstrate the feasibility of LL37 as a potential strategy in the reconstruction of clinical bone defects. Human adipose-derived mesenchyme stem cells (hADSCs) were cultured with different concentrations of LL37 and the optimum concentration for osteogenesis was selected for further *in vitro* studies. We then evaluated the antibiotic properties of LL37 at the optimum osteogenic concentration. Finally, we estimated the efficiency of a PSeD/hADSCs/LL37 combined scaffold on reconstructing bone defects in the rat calvarial defect model. The osteogenic ability on hADSCs *in vitro* was shown to be dependent on the concentration of LL37 and reached a peak at 4 μg/ml. The optimum concentration of LL37 showed good antimicrobial properties against *Escherichia coli* and *Staphylococcus anurans*. The combination scaffold of PSeD/hADSCs/LL37 showed superior osteogenic properties compared to the PSeD/hADSCs, PSeD, and control groups scaffolds, indicating a strong bone reconstruction effect in the rat calvarial bone defect model. In Conclusion, LL37 was shown to promote osteogenic differentiation *in vitro* as well as antibacterial properties. The combination of PSeD/hADSCs/LL37 was advantageous in the rat calvarial defect reconstruction model, showing high potential in clinical bone regeneration.

## Introduction

Bony orbital defects can be a result of trauma, malformations and iatrogenic surgery, and can lead to many functional and cosmetic problems that often require orbit reconstruction. Numerous types of biomaterials have been used to reconstruct the orbit including autogenous materials, metallic meshes, porous polyethylene, and resorbable biomaterials (Karsloğlu et al., 2006; Potter et al., 2012; Perez et al., 2018; Gao et al., 2020). Bioresorbable implants are becoming increasingly popular as they have several attractive properties including the ability to be easily shaped, strong mechanical integrity after resorption, and do not cause complications at the donor sites (Xuan et al., 2020).

Most bone defects can be reconstructed without the need to consider bacterial infections because they are isolated from the external environment. However, grafts in the bony orbital region are vulnerable to exposure and infections. In the bony orbit, grafts are often covered by thin scar tissues adjacent to the damaged paranasal sinus mucosa that contains a high number of bacteria (Ridwan-Pramana et al., 2015; Song et al., 2016). In comparison to solid sheet materials that are easily be encapsulated, bioresorbable implants are at higher risk of infection and inflammation. Once an infection occurs, patients are at risk of different complications including exposure, migration, and the formation of fistulae and cysts.

In cases where conservative treatment is unsuccessful, orbit reconstruction may fail and require implant removal. Presoaking implants with an antibiotic solution are recommended before the placement of implant materials. However, the antibiotic effect of presoaking implants is time-limited and some antibiotics have been reported to negatively impact bone regeneration (Wu et al., 2018). To avoid infection and also promote osteogenesis, there is a critical need for the development of novel engineered bone tissue materials for orbital defect reconstruction.

Bioactive scaffold-based tissue engineering has been widely investigated and applied as a strategy for bone regeneration (Deng et al., 2013; Orciani et al., 2017; Filippi et al., 2020; Shi et al., 2020). Poly(sebacoyl diglyceride) (PSeD) with exposed hydroxyl groups is a porous bioresorbable material which can be linked to bifunctional active molecules including growth factors, peptides, and chemical groups, making it a potential scaffold for multifunctional materials (You et al., 2012; Bi et al., 2014; Wang et al., 2016; Huang et al., 2016; Gong et al., 2020).

Antibacterial peptides (ABPs) have been explored as a potential compound with antibacterial and bone regenerating properties. The first ABP named “cecropin” was found in 1980 and since then major research efforts have focused on the purification and application of ABPs (Esfandiyari et al., 2019). ABPs can kill germs in multiple innate immune system models. ABPs found in mammals can be divided into two subtypes: defensins and cathelicidins.

LL37 is the only known human cathelicidin antimicrobial peptide (CAMP) and is formed from 37 amino acids of the C-terminus of the human cationic antimicrobial peptide-18 (Fan et al., 2015). LL37 contributes to host defenses against intracellular infections (Stephan et al., 2016) and the prevention of infection in burn care (Vignoni et al., 2014). LL37 can also cooperate with poly (lactic-co-glycolic acid) (PLGA) to accelerate wound healing (Chereddy et al., 2014) and can be used to treat keratitis caused by bacteria (Ishida et al., 2016). LL37 has shown much potential in hematopoietic stem/progenitor cells (HSPCs) and cord blood transplantation by accelerating the process of adhesion and recruitment (Wu et al., 2012). Recently, LL-37 has been combined with titanium implants to facilitate bone formation via mesenchymal stem cell recruitment (Liu et al., 2018; Shen et al., 2019). However, the effects of LL37 osteogenesis remain to be fully understood and there is a lack of knowledge on the use of LL37 in craniofacial bone defect reconstruction.

Most of the current studies have combined LL37 with bone mesenchymal stem cells (BMSCs) which are difficult to be harvested from human bone marrow. Compared to BMSCs, human adipose-derived mesenchymal stem cells (hADSCs) can be easily collected by liposuction or doubled eyelid surgery (Mehrabani et al., 2013). Moreover, ADSCs lack HLA-ABC expression and so ADSCs are less likely to be attacked by the host immune system due to the lack of HLA-ABC expression (Menard et al., 2013). hADSCs are therefore ideal seed cells for bone regeneration and could be widely applicable to many clinical indications.

In this study, we combined the biomaterial PSeD with hADSCs and the LL37 AMP and evaluated its effects on osteogenesis *in vitro* using *in vivo* rat calvarial bone defect model. The combination technique is expected to be applied in clinical surgery.

## Materials and Methods

### Preparation and Characterization of PSeD Scaffolds

Poly(sebacoyl diglyceride) (PSeD) was synthesized via acid-inducedepoxide ring-opening polymerization between an equimolar amount of diglycidyl sebacate and sebacic acid in presence of 0.6 mol % tetrabutylammonium bromide in dimethylformamide at 95°C for 24 h (You et al., 2010, 2012; Huang et al., 2016). The reaction mixture was purified via precipitation in ethyl ether and vacuum-dried to yield PSeD. The porous three-dimensional scaffolds of PSeD were prepared by a modified salt fusion template method using NaCl salt particles with a size of 75–150 mM as porogen according to previous reports (Huang et al., 2016; Sun et al., 2019; Gong et al., 2020; Xuan et al., 2020).

The mechanical properties of PSeD scaffolds were evaluated via compression tests as previous description (Gong et al., 2020). The morphology of PSeD scaffolds were investigated via scanning electron microscope.

### Cell Culture and Experimental Treatments

All research was approved by the Ethics Committee and the Animal Research Committee of Shanghai Ninth People’s Hospital, Shanghai Jiao Tong University School of Medicine, and informed consent was obtained from each subject. Human fat tissue was obtained from double eyelid surgery. Tissues were minced and digested with 0.1% collagenase I at 37°C with shaking at 200 rpm for 10 h. The released fat stromal cells were resuspended in Dulbecco’s modified Eagle’s medium (DMEM, Gibco) containing 10% fetal bovine serum (FBS, Gibco) and 100 units/ml of penicillin and streptomycin (Invitrogen) before incubation at 37°C in an atmosphere of 5% CO_2_. ADSCs were characterized by flow cytometry (CD44, CD90, CD73, CD45, CD34 and HLA-DR, all from BD Biosciences, San Jose, CA, United States), *in vitro* adipogenic and chondrogenic induction assays. To investigate the optimum concentrations of LL37 with the strongest osteogenic effect, after incubation for 24 h, different concentrations of LL37 (0, 1, 2, 4, 8 μg/mL) were separately added to the culture medium.

### *In vitro* Cytotoxicity

The effects of LL37 on the proliferation of hADSCs were evaluated using a cell counting kit-8 (CCK-8) assay as previously reported (Ni et al., 2014). Cells were seeded at a density of 3.0 × 10^4^/cm^2^ in a 96-well plate and incubated with different concentrations of LL37 (0, 1, 2, 4, 8 μg/mL). 10 μl of CCK-8 solution was added to each well (Dojindo Molecular Technologies, Inc., Japan) incubated at 37°C for 4 h. The absorbance values at 450 nm wavelength were measured.

### Detection of Osteogenic Activity *in vitro*

#### Real-Time Reverse Transcription-Polymerase Chain Reaction (RT – PCR)

After 7 days of culture, the total RNA of hADSCs was collected and purified using the EZ-press RNA purification kit (EZBioscience). Reverse transcription was performed using the PrimeScript RT reagent kit (Takara, Dalian, China). Quantitative RT-PCR was optimized with a Power SYBR Green PCR Master Mix (Applied Biosystems, Foster City, CA, United States) using a 7500 Real-Time PCR detection system. The related primer sequences are listed in Table 1 including collagen I (Col1), osteopontin (Opn), runt-related transcription factor 2 (Runx2), bone sialoprotein (BSP), and GAPDH. Relative mRNA levels were normalized to the expression level of GAPDH. All tests were performed in triplicates.

**TABLE 1:** Real-time polymerase chain reaction primers used in this study.

**Genes**	**Forward (5′-3′)**	**Reverse (5′-3′)**
COL1	GAGGGCCAAGACGAAGACATC	CAGATCACGTCATCGCACAAC
OPN	CTCCATTGACTCGAACGACTC	CAGGTCTGCGAAACTTCTTAGAT
Runx2	CAGTAGATGGACCTCGGGAACC	GGCGGGACACTACTCTCATAC
BSP	CCCCACCTTTTGGGAAAACCA	TCCCCGTTCTCACTTTCATAGAT

#### Western Blotting

After culturing hADSCs in different concentrations of LL37 for 7 days, RIPA (Cell Signaling Technology) and loading buffers (Takara) were used to lyse hADSCs. An SDS-PAGE gel (12%) was prepared and used for the electrophoretic separation of the proteins at 80 V for 30 min, and then 120 V for 30 min. After electrophoresis, the gel was transferred to a 0.22 μm polyvinylidene fluoride (PVDF) membrane (Millipore, Billerica, MA, United States) and blocked in 5% BSA for 1 h at room temperature. The PVDF membrane was incubated with primary antibodies against Col1, Opn, Ocn, Runx2, Bsp (Santa Cruz Biotechnology, Inc.), and β-actin (Abcam, Cambridge, MA, United States) overnight at 4°C. TBST buffer was used to wash the PVDF membrane three times for 10 min before the membranes were probed with peroxidase-conjugated affinipure goat anti-mouse IgG (H + L) (Jackson ImmunoResearch Laboratories, Inc., United States). Finally, the membrane was scanned using a Tanon-5200 image scanner (Tanon, China) and the relative gray levels of proteins were normalized by the β-actin gray level.

#### Alkaline Phosphatase Staining

After culturing in different concentrations of LL37 for 7 days, hADSCs were fixed in 4% paraformaldehyde and stained with alkaline phosphatase (ALP) staining buffer avoiding light for 2 h at room temperature as previously described (Deng et al., 2014). The staining results were observed using an optical microscope (Nikon) and analyzed by NIS-Elements Viewer 4.50.

#### Osteogenic Effect of the Combination of PSeD and LL37

The two-dimensional crosslinked PSeD materials were cultured with cells and LL37 to detect the osteogenic potential of the PSeD and LL37 combination. The human ADSCs were culture in three groups: the plain plate (control), the slide coated by (PSeD) and the PSeD combined with LL37 group (PSeD + LL37). The concentration of LL37 was determined by previous work. ALP staining was performed as described before after culturing for 7 days.

### Antibiotic Testing

The optimum concentration of LL37 for osteogenic effects was determined according to the previous procedure. Three methods were used to evaluate the antimicrobial properties of LL37 in the optimum osteogenic concentration including gross antibacterial activity, inactivity efficiency detection, and live-dead staining for both Gram-negative and Gram-positive bacteria. Gross antibacterial activity was determined using 10^7^ colony-forming units (CFU) in Gram-negative bacteria *Escherichia coli* and Gram-positive bacteria *Staphylococcus anurans.* These strains were separately added to the optimum concentration of LL37 in broth and then vibrated on a table concentrator at 37°C for 6 h. 100 μL of bacteria solution was seeded on the plate at 37°C overnight. The colonies on the plate were recorded as the results of gross antibacterial activity. For inactivity efficiency detection, bacterial solutions were diluted 100 times with broth and transferred into a 96-well plate. The OD600 nm values were measured every 30 min for 6 h. After 6 h, the remaining bacterial solution in the 96-well plate was dyed with live-dead dyeing buffer for 15 min for the remaining bacteria measured.

### Preparation of Material for *in vivo* Experiments

PSeD scaffolds with 5 mm in diameter were prepared and sterilized using an autoclave sterilizer and then placed in a 24-well plate using sterilized tweezers. From the *in vitro* experiments, an adequate LL37 concentration for antibacterial and osteogenic effects was determined to be 4 μg/mL. This concentration was used to culture hADSCs in six-well plates (2.5 × 10^6^ hADSCs/2 mL/well). For each 5-mm diameter bone defect and the detection of subcutaneous biocompatibility, the cell load of the scaffold was approximately 5 × 10^6^ and the required amount of LL37 added to each scaffold of 5 mm in diameter was 8 μg.

### *In vivo* Experiments

#### *In vivo* Biocompatibility Detection

A total of six 8-week-old female Sprague Dawley rats were used in this study. The animals were anesthetized and all surgical procedures were performed under sterile conditions. Four different subcutaneous dorsum pouches were created on each rat. Each animal received three different implants and a control pouch is as follows: blank control, pure PSeD implant, PSeD implant with hADSCs (5 × 10^6^ cells), PSeD implant with hADSCs (5 × 10^6^ cells), and LL37 (8 μg). Two weeks after surgery, animals were euthanized by general anesthesia, the pouches were harvested with the implanted materials and then fixed in 4% paraformaldehyde. The samples were cut into 8 μm sections before hematoxylin and eosin (H&E) staining. Immunofluorescence staining was performed for macrophage-related markers (CD80 for M1 and CD68 for M2 type macrophages) to detect the implant peripheral inflammatory response.

#### Detection of Osteogenic Effects *in vivo*

To determine the *in vivo* osteogenic effects of the combination of PSeD/human-ADSCs/LL37, we fabricated 12 rat calvarial defect models. This involved the generation of a 5-mm diameter defects drilled using a dental trephine (5 mm in external diameter) (Nouvag AG, Goldach, Switzerland) into both sides of the left and right halves of the calvarium. To repair the defect, three types of implants were embedded into the defects of every four rats, including pure PSeD, PSeD/hADSCs, PSeD/hADSCs/LL37. The rest four rats were implanted nothing as the control group.

Sequential fluorescent labeling of the newly formed mineralization was performed after surgery according to previous reports (Ding et al., 2017). Fluorochromes including tetracycline (25 mg/kg of body weight), Alizarin Red (30 mg/kg of body weight), and calcein (20 mg/kg of body weight, from Sigma) were administered to the animals by intraperitoneal injection at 2, 4, and 6 weeks of post-surgery.

### Morphological and Histological Evaluation

Eight weeks after the surgery, rats were culled by overdose of anesthesia and death confirmed by cervical dislocation. The skulls were removed and soaked in 4% paraformaldehyde. Image analysis was performed by micro-CT (Bruker SkyScan1076) in the coronal and sagittal planes using previously reported imaging parameters (Deng et al., 2014) as follows: tube current (250 μA), X-ray tube potential (40 kV), a region of interest (5 mm diameter round), voxel resolution (35 mm). The percentage of bone volume (bone volume/tissue volume, BV/TV) and bone mineral density (BMDs) of the bone defect, were detected using the MicroView (GE Healthcare, Waukesha, WI, United States) software package.

After scanning for morphological defects, skulls were embedded in polymethylmethacrylate and dehydrated through an alcohol gradient. The embedded skulls were cut into 300 μm coronal sections using a microtome (EXAKT310, Germany). Sections were photographed under different luminescence wavelengths (tetracycline at 405 nm, calcein at 488 nm, and alizarin red at 543 nm). Images were acquired using a Nikon A2 confocal camera.

### Statistical Analysis

All the data in this study are presented as the mean ± SD. Unpaired Student’s *t*-test was used for statistical analysis. *P* < 0.05 was set to determine statistically significant differences.

## Results

### Characterization of PSeD Porous Scaffolds

The results of compression tests and scanning electron microscope imaging was shown in the Supplementary Figure 1.

### Viability of hADSCs

**FIGURE 1:**
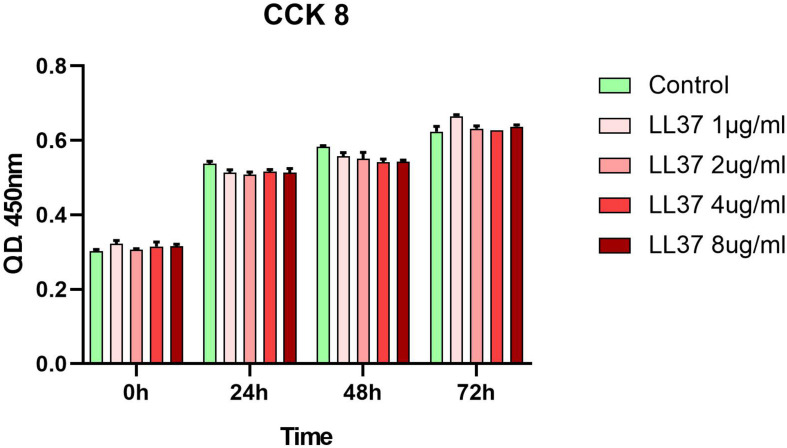
Effects of LL37 on the proliferation of human ADSCs. Cellular viability was evaluated using a cell counting kit-8 (CCK-8) assay and analyzed with the OD450 nm value (*n* = 3, unpaired *t*-test).

Human ADSCs, were identified by flow cytometry results and showed high expression of CD90, CD44, and CD73, with lack expression of CD45, CD34, and HLA-DR. Their capacities for differentiating into chondrocyte and adipocyte lineages were proved after *in vitro* adipogenic and chondrogenic induction (Supplementary Figures 2–4). The CCK8 (Figure 1) assay revealed that hADSCs cultured with different concentrations of LL37 had similar proliferation responses to the control group. These data indicated that low concentrations of LL37 had no cytotoxic effects on hADSCs.

### LL37 Promotes Osteogenic Differentiation of hADSCs *in vitro*

**FIGURE 2:**
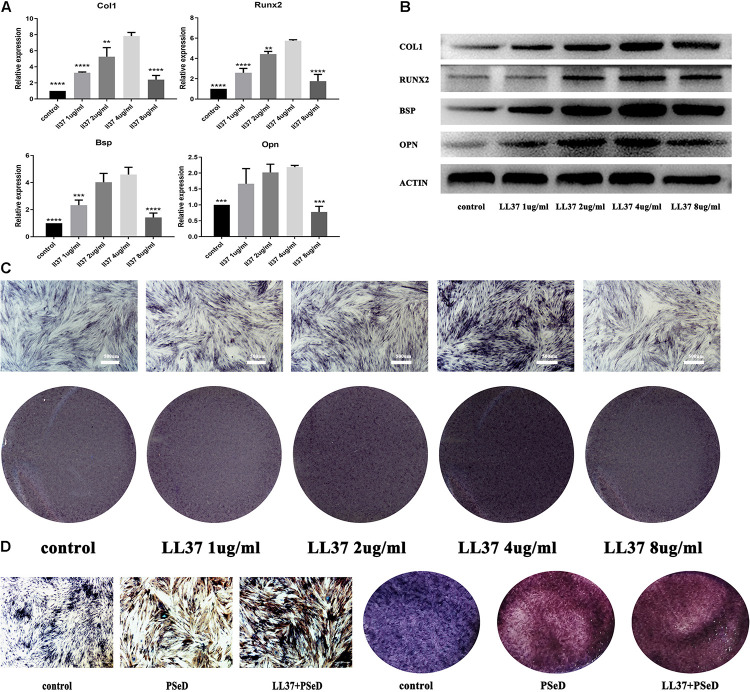
Effects of LL37 on the osteogenic expression levels of human ADSCs in normal DMEM. Human ADSCs were cultured separately in the control group using DMEM. The experimental groups used different concentrations of LL37 (1, 2, 4, 8 μg/mL). **(A)** RNA was harvested on day 7. PCR results showed the expression level of mRNA (Col1, Opn, Runx2, Bsp) reached a peak at 4 μg/mL concentration (*n* = 3, compared to 4 μg/mL concentration group, unpaired *t*-test, ^*^ *p* < 0.05, ^**^ *p* < 0.01, ^***^ *p* < 0.001, ^****^ *p* < 0.0001). **(B)** Proteins were extracted on day 7. Western blotting results suggested that 4 μg/mL of LL37 was most effective in promoting osteogenic protein expression (Col1, Opn, Runx2, Bsp). **(C)** Results of ALP on the 7th day also supported the protein and RNA expression data. **(D)** ALP staining results indicated that the combination of PSeD and LL37 can effectively promote osteogenesis.

To evaluate the capacity of LL37 to accelerate osteogenic differentiation, different concentrations of LL37 were added to the hADSCs inoculum. At day 7, RNA was harvested and RT-PCR was performed as described. The results (Figure 2A) showed that hADSCs cultured with low concentrations of LL37 had significantly upregulated expression levels of related genes that reached a peak at the concentration of 4 μg/ml. However, amongst all the concentrations investigated, 8 μg/ml had no significant increases in the mRNA expression of Runx2, Bsp, COL1, and Opn compared to the control group. Western blotting was performed on samples obtained at day 7 (Figure 2B) to determine the levels of Runx2, Bsp, COL1, and Opn proteins. Our data showed that the group with 4 μg/ml of LL37 exhibited the highest level of osteogenic protein expression. ALP results (Figure 2C) indicated that over time, medium containing 4 μg/mL of LL37 had the greatest effect on promoting cell osteogenic differentiation. ALP staining results grossly and microcosmically also indicated that the combination of PSeD and 4 μg/mL LL37 can effectively promote osteogenesis (Figure 2D).

### Antibacterial Activities of LL37 in Gram-Negative and Gram-Positive Bacteria

**FIGURE 3:**
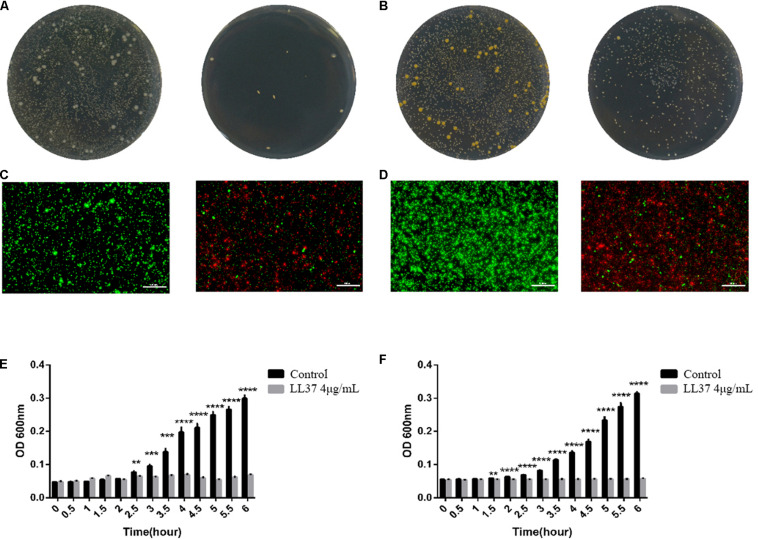
Antibacterial effects and inactivity efficiency of 4 μg/mL of LL37 against *E. coli* and *S. aureus.* 10^4^ CFU bacteria were cultured in 4 μg/mL of LL37 and PBS of the same volume (control group) for 6 h. **(A)** Gross antibacterial results showed the colony number of *E. coli* treated with LL37 was significantly smaller than that of the control group. **(B)** Gross antibacterial results indicated that the colony number of *S. aureus* treated with LL37 was significantly smaller than that of the control group. **(C)** The results showed that the number of live *E. coli* treated with LL37 was less than that of the control group, but the number of dead bacteria was greater than that of the control group. **(D)** The results implied that the number of live *S. aureus* treated with LL37 was less than that of the control group, but the number of dead bacteria was greater than that of the control group. **(E)** The OD600 nm value of bacteria treated by LL37 and PBS (control) against *E. coli* showed a significant difference between the LL37 group and the control group after 2.5 h. The OD value of the LL37 group did not fluctuate significantly within 6 h which proved that the activity of bacteria was significantly affected. The bacterial proliferation of the LL37 group was at a standstill whilst bacterial proliferation was obvious in the control group. **(F)** Tracing OD600 nm value of bacteria treated by LL37 and PBS (control) against *S. aureus* showed a significant difference between the LL37 group and the control group after 1.5 h. The OD value of the LL37 group did not fluctuate significantly within 6 h, which proved that the activity of bacteria was significantly affected. The bacterial proliferation of the LL37 group was at a standstill, while the bacterial proliferation was obvious in the control group. The scale bar is 100 μm (*n* = 3, unpaired *t*-test, ^**^ *p* < 0.005, ^***^ *p* < 0.0005, ^****^ *p* < 0.0001).

As the optimum concentration for osteogenic differentiation was determined previously, the antimicrobial properties of LL37 were confirmed at 4 μg/mL using the three methods. Gross antibacterial activities showed reduced colonies on the plate with LL37 compared to controls. These data indicated that 4 μg/mL of LL37 was effective in killing both Gram-negative (Figure 3A) and Gram-positive bacteria (Figure 3B). Live-dead staining results also suggested that 4 μg/mL of LL37 can effectively kill the *E. coli* (Figure 3C) and *Staphylococcus aureus* (Figure 3D). The results of the inactivity efficiency detection showed significantly lower values in the LL37 group after 3.5 h when seeded with each of the bacteria (Figures 3E,F).

### *In vivo* Biocompatibility

**FIGURE 4:**
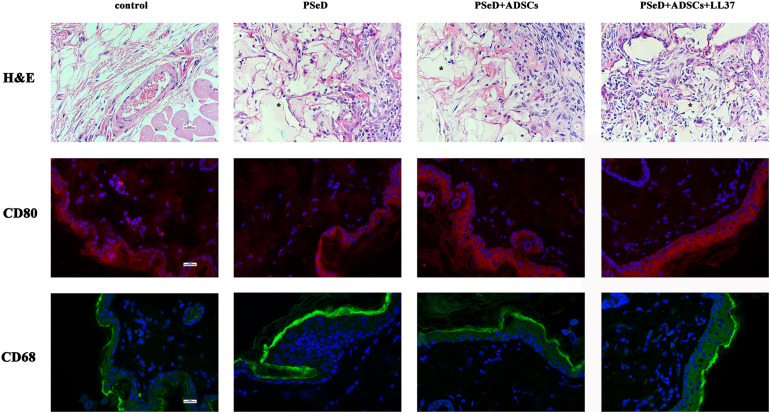
Representative photomicrographs of H&E staining sections and immunofluorescence staining for CD68/CD80 positive macrophages. **First row:** H&E staining, PSeD is shown as pink-glass-like opacities and was marked with ^*^. **Second and third rows:** Immunofluorescent staining of macrophage-specific markers, CD80 for M2 and CD68 for M1. Each row contains photos of four groups: (1) control group with no implant; (2): PSeD; (3): PSeD + ADSCs; (4): PSeD + ADSCs + LL37. There was no significant difference amongst the four groups. All photos were taken at 400 times magnification. The scale bar is 25 μm.

Hematoxylin and eosin (H&E) staining (Figure 4) suggested that the surrounding soft tissue in all groups had a normal histological structure. Immunofluorescence staining for CD80 (labeled by red fluorescence in the cytoplasm, Figure 4) was used to estimate M1 macrophage activation. CD68 (labeled by green fluorescence in the cytoplasm, Figure 4) was adopted to identify M2 macrophage activation triggered by implantation. The distributions of newly recruited (CD-80 and CD68 positive) subcutaneous macrophages in the implantation groups exhibited similar patterns compared to the control group which validated the observations from the H&E staining.

### The Combination of PSeD/hADSCs/LL37 Promotes Bone Regeneration in Calvaria Defect of Rats

**FIGURE 5:**
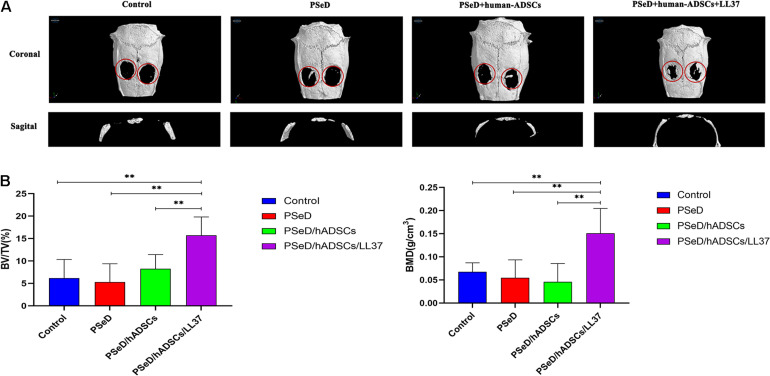
Micro CT and analysis results of rat calvarial bone defect reconstruction. **(A)** Micro CT results: the reconstruction area increased from left to right: control group, PSeD group, PSeD/hADSCs group, PSeD/hADSCs/LL37 group. **(B)** Micro CT statistical analysis results: compared with the other three groups, the PSeD/hADSCs/LL37 group performed better in bone volume/tissue volume (BV/TV) ratio and bone mineral density (BMD) with significant differences (*n* = 6, unpaired *t*-test, ^∗∗^ *p* < 0.01).

**FIGURE 6:**
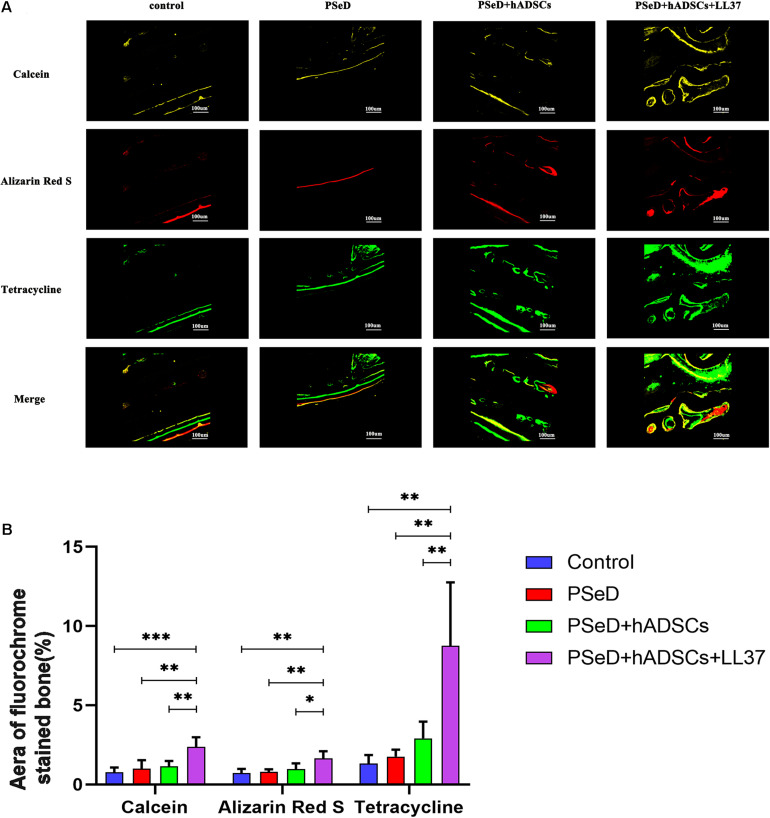
Fluorescence labeling results in new bone formation. **(A)** Confocal microscope images for each group. **Row 1** (yellow) represents new bone formation marked by calcein injected at week 6, **row 2** (red) represents alizarin red at week 4, **row 3** (green) represents tetracycline at week 2, and **row 4** represents merged images of the three fluorochromes for the same group. **(B)** The graph shows the percentages of each fluorochrome area for the different groups. Significant differences were observed between PSeD/hADSCs/LL37 group and PSeD/hADSCs group (*n* = 6, unpaired *t*-test, ^∗^ *p* < 0.05, ^∗∗^ *p* < 0.01, ^∗∗∗^ *p* < 0.001).

Our *in vitro* results provided preliminary indications that LL37 promotes osteogenic differentiation. To explore the effect of LL37 on bone defects *in vivo*, we observed the new bone formation and quantified the regenerated bone in PSeD/hADSCs/LL37, PSeD/hADSCs, PSeD, and NC composites using the rat calvarial bone defects by high-resolution micro-CT scanning. As shown in Figure 5A, calvarial bone defects treated with the PSeD/hADSCs/LL37 composite showed the highest level of new bone formation compared to the other three groups. Quantitative morphometric analysis using micro-CT showed that the bone volume/tissue volume (BV/TV) ratio in the PSeD/hADSCs/LL37 group (15.72 ± 3.74%) was significantly higher than those of the other three groups (8.27 ± 2.89%, 5.30 ± 3.74%, and 6.17 ± 3.80% in the PSeD/hADSCs, PSeD, and NC groups, respectively) (*P* < 0.05) (Figure 5B). The BMD showed the same pattern as the BV/TV ratio (0.1507 ± 0.0491 g/cm^3^, 0.0458 ± 0.0362 g/cm^3^, 0.0545 ± 0.0355 g/cm^3^, and 0.0674 ± 0.0178 g/cm^3^ in the PSeD/hADSCs/LL37, PSeD/hADSCs, PSeD, and NC groups, respectively) (*P* < 0.05) (Figure 5B). The early, mid and late stages of bone mineralization were labeled by calcein, alizarin red and tetracycline fluorescence, respectively, and quantified (Figure 6). The luminogram of tetracycline, calcein, alizarin red indicated similar trends of new bones, suggesting the superiority of the PSeD/hADSCs/LL37 combination.

## Discussion

LL37 exerts multiple biological effects on tissues including wound healing, immune responses and neovascularization in injured tissues. Recent studies have suggested that LL37 can facilitate bone repair in a femur defect model by promoting the recruitment of BMSCs. However, the ability of LL37 to enhance bone regeneration by cell osteogenic differentiation (especially, in calvarial defect model) has not yet been determined. For the first time, in this study, we evaluated the combination of LL37 and hADSCs in the treatment of rat calvarial defect model. Our results show that a combination of LL37 and hADSCs promoted bone regeneration and inhibited the inflammations after implantation.

*In vivo* and *in vitro* biocompatibility are crucial factors in the success of bone reconstruction. Anders et al. (2018) reported that high concentrations of LL-37 reduced human osteoblast-like MG63 cell viability *in vitro*. These findings highlight the importance of determining the cytotoxic effects of LL37 on bone regeneration cells. In this study, The ADSC proliferation rate was measured using a CCK-8 assay. Compared with the no substrate group, low concentrations of LL37 had no cytotoxicity on hADSCs *in vitro*. We detected the peripheral inflammatory response in rats after implantation of PSeD combined with LL37/hADSCs. Our results indicated that the surrounding soft tissue had a normal histological structure and host response to the implants in all the experimental groups. We then verified that low concentrations of LL37 combined with PSeD/hADSCs have good biocompatibility *in vivo*, which would be applicable in tissue reconstruction.

The osteogenic effect of LL37 has previously been reported, however, most studies have focused on stem cell recruitment and the combination of LL37 with bone marrow stem cells (BMSCs) (Kittaka et al., 2013). Although BMSCs have been shown to exhibit differentiation potential, the number of BMSCs that can be obtained from a single bone marrow aspirate is limited. Therefore, *in vitro* culture expansion is necessary which results in inevitable loss of potency (Dhinsa and Adesida, 2012). Recently, studies have focused on ADSCs as an alternative autologous source of BMSCs that are less controversial and have fewer limitations compared to embryonic stem cells and BMSCs (Patrikoski et al., 2019). However, it still remains unclear whether LL37 could promote osteo-differentiation and osteogenesis on ADSCs.

ADSCs have major potential in regenerative medicine and as they contain thousands of times more stem cells per unit volume than bone marrow aspirates (Xie et al., 2012; Marolt Presen et al., 2019). The separation of ADSCs by differential centrifugation in the operating room makes ADSCs a viable candidate for any stage of the procedure. Our data indicated that LL37 plays an important role in the induction of hADSC osteogenic differentiation. Compared to the control group, LL37-treated hADSCs exhibited higher expression levels of osteogenic-related genes. Furthermore, the effect of LL37 was concentration- dependent with 4 μg/mL showing the highest expression level of osteogenic-related genes. To further confirm osteogenic differentiation at the protein level, the levels of osteogenic-related proteins (BSP, RUNX2, and OPN) were determined. Our data showed a close similarity between the mRNA and protein expression levels. In addition, ALP, a widely expressed enzyme in the bone responsible for the mineralization of new bone, had higher expression in LL37 group compared to the control group. These findings were consistent with the mRNA expression levels and indicated that LL37 enhanced hADSC osteogenic differentiation. The combination ofLL37 and PSeD also showed good effect on enhancing hADSC osteogenic differentiation.

Traditional conservative treatment of orbital infection includes local antibiotics and intravenous administration. Antibiotic resistance is becoming an increasing problem that is associated with strains of bacteria such as MRSA and so there is an urgent need for the development of new antibacterial substances such as AMPs (Hou et al., 2013; Fabisiak et al., 2016). However, the safe and effective use of peptides remains to be fully determined. In this study, we confirmed the antibacterial and inactivity efficiency of LL37 at a concentration of 4 μg/mL, which was optimal for osteogenic differentiation. The concentration also coincided with the work of Noore et al. (2013), who reported a 90% killing rate at 250 nM (about 4.8 μg/mL) LL37 on *S. aureus*. Infections in the bone involve multiple pathogens including Gram-negative *E. coli* and Gram-positive *S. aureus* (Murray et al., 2011). In this study, the gross antibacterial activity, the inactivity efficiency detection and the live-dead staining results all showed high efficacy in the treatment of *S. aureus* and *E. coli* indicating a potential role in preventing bone infections.

Therefore, based on the osteogenic differentiation LL37/PSeD on hADSCs *in vitro*, good biocompatibility of PSeD/hADSCs/LL37 *in vivo*, we further investigated its effects on *in vivo* bone regeneration. Our *in vivo* bone regeneration experiments used rat calvarial bone defect model which differs from the more commonly used femur defect model (He et al., 2018; Shen et al., 2019). We applied LL37 accompanied with PSeD/hADSCs and showed the ability of bone regeneration without stress stimulation, which is similar to that of the bony orbit. These results suggested that the combination of PSeD/hADSCs/LL37 can significantly accelerate the process of bone reconstruction. Quantitative analysis revealed that PSeD/hADSCs/LL37 was also superior to others in terms of BV/TV and BMD. In addition, fluorochrome-labeling using calcein, alizarin red and tetracycline at the early, mid and late stages of bone regeneration indicated that LL37 could continuously promote the formation of new bone. Given that the combination of PSeD/hADSCs/LL37 could promote bone regeneration *in vivo* in the rat calvarial bone defect model, LL37 may be a promising therapeutic option for bone regeneration, especially in sites without stress stimulation, such as the orbit.

Our study had a number of associated limitations. Although we showed that LL37 could promote bone regeneration while also enhancing hADSC osteogenic differentiation, the underlying mechanisms of these processes remain unclear. The relationship between hADSCs osteogenic differentiation effects and the concentrations of LL37 were only primarily determined. Further investigations are needed to better elucidate the mechanism of LL37 in the reconstruction of bone defects.

## Conclusion

In summary, LL37 was shown to promote hADSCs osteogenic differentiation in a concentration-dependent manner. We found that the optimum concentration *in vitro* was 4 μg/mL which was effective in the bacteriostasis for both Gram-positive and Gram-negative bacteria. The combination of PSeD/hADSCs/LL37 promotes osteogenic differentiation *in vitro* and bone regeneration *in vivo* in the rat calvarial bone defect model. In conclusion, the osteogenic function and antibacterial ability of low concentrations of LL37 suggest it may be promising for orbit bone defect reconstruction and future clinical applications.

## Data Availability Statement

The raw data supporting the conclusions of this article will be made available by the authors, without undue reservation.

## Ethics Statement

The studies involving human participants were reviewed and approved by the Ethics Committee of Shanghai Ninth People’s Hospital, Shanghai Jiao Tong University School of Medicine. The patients/participants provided their written informed consent to participate in this study. The animal study was reviewed and approved by Animal Research Committee of Shanghai Ninth People’s Hospital, Shanghai Jiao Tong University School of Medicine.

## Author Contributions

AZ and XB: conception of the study. LL and YP: manuscript preparation. LL, YP, QY, and JS: experimental work and data analysis. AZ and XB: supervision. All authors contributed to the article and approved the submitted version.

## Conflict of Interest

The authors declare that the research was conducted in the absence of any commercial or financial relationships that could be construed as a potential conflict of interest.
