# Supplementary material for: Cathelicidin LL37 Promotes Osteogenic Differentiation in vitro and Bone Regeneration in vivo
Source: Front Bioeng Biotechnol. 2021 May 3;9:638494. doi: 10.3389/fbioe.2021.638494 (PMC8126666; doi:10.3389/fbioe.2021.638494)
Supplement: Supplementary file 1 [file Data_Sheet_1.pdf]

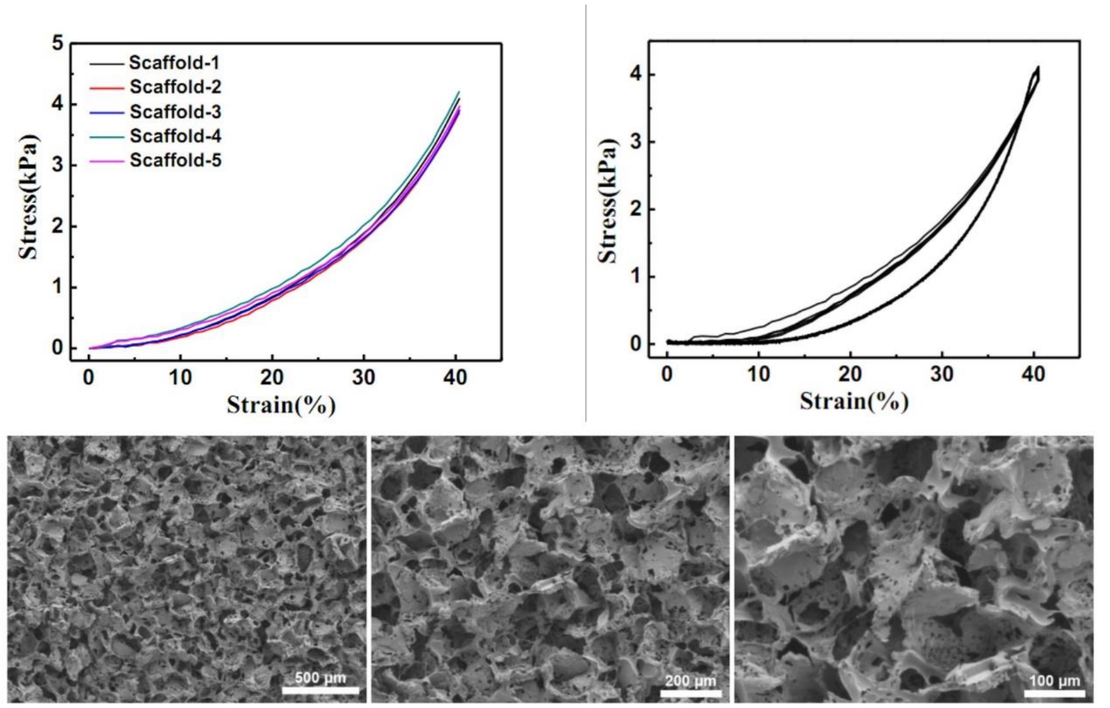

Figure 1. Tensile compression results and scanning electron microscope images of porous PSeD scaffolds. Top left is the result of single compression tests. Top right is the result of cyclic compression tests. Scanning electron microscope images are in the bottom.

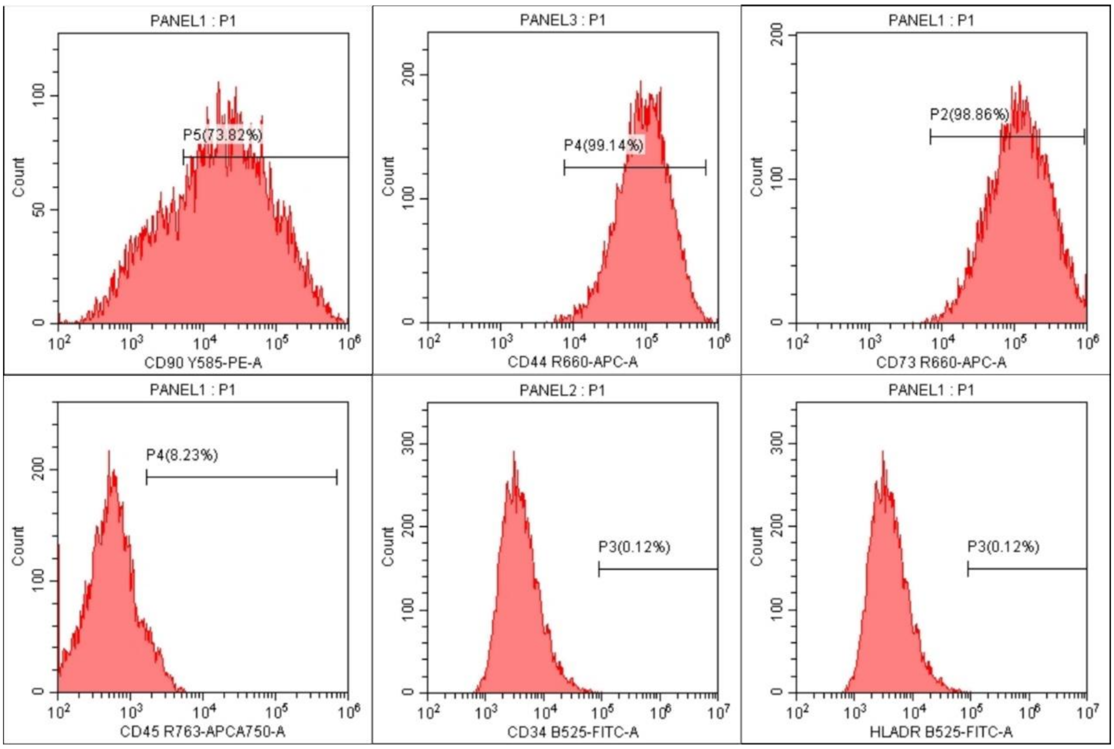

Figure 2. Flow cytometry results. Cells express high proportion of CD90, CD44 and CD73, with lack expression of CD45,CD34 and HLA-DR.

Human MesenCult™ Adipogenic Differentiation Medium (Stemcell) was used for adipogenic induction as proposals. Cells were plated in 2 mL of growth medium per well and incubated at 37°C until they are approximately 90 - 100% confluent. Medium was replaced with 2 mL of complete MesenCult™ Adipogenic Differentiation Medium per well. Then, cells were incubated at 37°C and medium was changed every 3 days. After 14 days of culturing, lipid vacuoles were observed by Oil Red O staining.

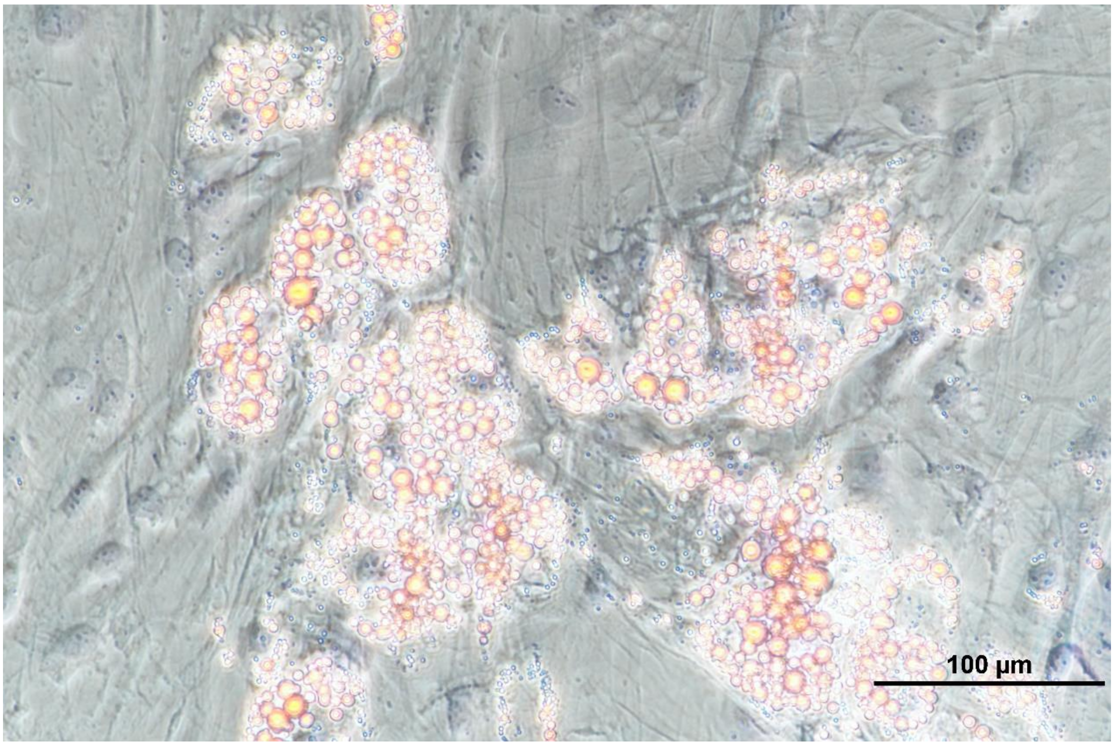

Figure 3. Lipid vacuoles stained by Oil Red O were observed in high magnification.

Chondrogenic induction kit were purchased from Cyagen Biosciences Inc. (Guangzhou, China) . About 40,000 cells werecultured in 15 mL Centrifuge tube with chondrogenic induction medium. Then, cells were incubated at 37°C and medium was changed every 3 days. About 28 days, cartilage ball was fixed and stained by Alcian blue. Endo acidic mucopolysaccharide in cartilage was visualized.

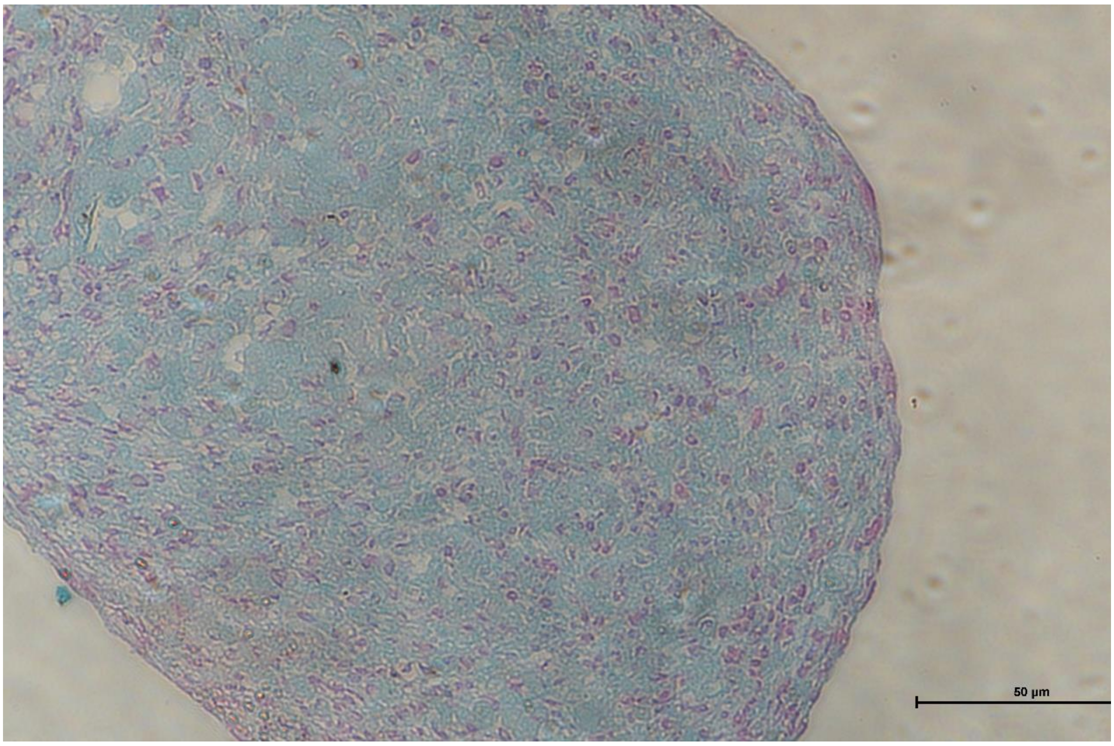

Figure 4. Cartilage stained by Alcian blue were observed in high magnification.
